# Healthcare Professionals' Attitude to Using Mobile Health Technology and Its Associated Factors in a Resource-Limited Country—An Implication for Digital Health Implementers: A Cross Sectional Study

**DOI:** 10.1155/2024/1631376

**Published:** 2024-07-12

**Authors:** Agmasie Damtew Walle, Fikadu Wake Butta, Sisay Yitayih Kassie, Alex Ayenew Chereka, Shuma Gosha Kanfe, Abiy Tasew Dubale, Ermias Bekele Enyew, Geleta Nenko Dube, Adamu Ambachew Shibabaw, Mekonnen Kenate Hunde, Gemeda Wakgari Kitil, Tigist Andargie Ferede, Sisay Maru Wubante, Nebebe Demis Baykemagn, Addisalem Workie Demsash

**Affiliations:** ^1^ Department of Health Informatics School of Public Health Asrat Woldeyes Health Science Campus Debre Berhan University, Debre Birhan, Ethiopia; ^2^ Department of Health Informatics College of Health Science Mattu University, Metu, Ethiopia; ^3^ Department of Health Informatics School of Public Health Hawassa University, Hawassa, Ethiopia; ^4^ Department of Health Informatics School of Public Health College of Health Science Wollo University, Dessie, Ethiopia; ^5^ Department of Lifelong Learning and Community Development College of Education and Behavioral Science Mattu University, Metu, Ethiopia; ^6^ Department of Midwifery College of Health Science Mettu University, Metu, Ethiopia; ^7^ Department of Epidemiology Institute of Public Health College of Medicine and Health Science University of Gondar, Gondar, Ethiopia; ^8^ Department of Health Informatics Institute of Public Health College of Medicine and Health Science University of Gondar, Gondar, Ethiopia

**Keywords:** attitude, Ethiopia, healthcare professionals, mHealth

## Abstract

**Background:** Mobile health has become widely used within the healthcare system, and there is an increasing worldwide trend toward employing this innovation for behavior management, disease monitoring, the control and prevention of various health issues, and rising enrollment in healthcare services. Although mHealth is becoming more widely available, there is no evidence about the attitude of healthcare professionals toward mHealth in southwest Ethiopia. Therefore, this study is aimed at assessing the attitude of healthcare professionals to using mHealth technology and associated factors in Ethiopia.

**Methods:** An institutional cross-sectional study was conducted among 422 healthcare professionals. Data were collected using a pretested interviewer-administered questionnaire, and the study was conducted from January 08 to February 10, 2023. EpiData Version 4.6 for entering the data and STATA Version 14 for analyzing the data were used. A multivariable logistic regression analysis was carried out to identify factors associated with healthcare professionals' attitudes to using mobile health technology.

**Results:** A total of 415 study participants were included in the study. About 180 (43.4%) respondents had a favorable attitude toward mHealth technology in southwest public hospitals. Master's degree and above (adjusted odds ratio [AOR]: 3.67; 95% CI: 1.22, 4.10), good knowledge of mobile health technology (AOR: 4.08; 95% CI: 1.35, 5.31), more than 5 years of work experience (AOR: 3.09; 95% CI: 1.76, 5.60), had ICT infrastructure (AOR: 2.70; 95% CI: 1.38, 5.31), had own smart mobile (AOR: 3.67; 95% CI: 3.20, 4.31), and had taken computer-related training (AOR: 1.96; 95% CI: 1.03, 3.73) were positively associated with healthcare professionals' attitude to using mobile health technologies in southwest Ethiopia.

**Conclusions:** Overall, healthcare professionals' attitude to using mobile health technologies in southwest Ethiopia was relatively low. Education level, good knowledge, years of work experience, ICT infrastructure, having a smart mobile, and having taken computer-related training were significant factors of attitude to using mobile health technologies. Considering these factors could provide insight into developing and adopting mobile health technologies in Ethiopia.

## 1. Introduction

The WHO established a global digital health strategy for 2020–2025 in 2020 [1]. Digital health technology like mHealth adoption is changing the exploration, advancement, and provision of health-related products and services, as well as the identification, management, and protection of health conditions [2]. These advancements are laying the groundwork for reasonably priced, available, and high-quality medicines, vaccines, medical devices, and system innovations, as well as seeking innovative concepts and efforts to solve the most difficult health problems [3]. The application of mobile technology to provide healthcare services is recognized as mobile health [4]. According to the WHO, mHealth has the potential to promote the health system and potentially trigger a fundamental change in healthcare procedures around the world [5]. Mobile health has become widely used within the healthcare system, and there is an increasing worldwide trend toward employing this innovation for behavior management, disease monitoring, the control and prevention of various health issues, and rising enrollment in healthcare services [6]. As a consequence, the concept of mobile health has indeed been considered a possible remedy to several challenges facing low-income countries [7].

Mobile-based health technology has the potential to substantially reduce the cost of healthcare for impoverished people in resource-limited countries [2]. The intensifying rapid spread of mobile phones offers the possibility of using mobile devices for health initiatives [3]. It can empower individuals by offering valuable and efficient information that enhances and reduces barriers to care and service delivery while attempting to address the shortage of healthcare workers [8]. Text messaging, voice calling, media platforms like WhatsApp and Twitter, and emails are all ways to share information via mobile phone [9]. It serves as a vehicle for providing information regarding the characteristics, comorbidities, and problems of a certain health condition [10]. Mobile phones have become increasingly common in disease prevention and follow-up intervention strategies in low-income countries, where they are being used to implement actions to promote a healthy diet, regular physical activity, and obesity prevention and increase patient engagement in healthcare [11].

People living in resource-constrained countries, such as numerous African countries, are at a greater risk of several health issues than those residing in other regions, despite having the least access to health innovations [12]. Africa's countries cannot meet their population's health demands and requirements unless urgent technological, industrial, intellectual, and evidence-based measures are put in place [12]. Health technology innovations are required to reduce inequalities in Africa's health systems, and it is critical to generate a region-based approach to determine difficulties and opportunities in the region as potential resources for future interventions [13].

Nearly half (48.5%) of doctors in Uganda had a good attitude toward eHealth [13]. A study conducted in Kenya revealed that 73.53% of nurses had a favorable attitude toward mobile health applications and computerization [14]. Similarly, a study done in Legos showed that most doctors (95.4%) and nurses (88.9%) had favorable attitudes toward mHealth technology [15].

Despite all of these efforts, the Ethiopian Demographics Health Survey (EDHS) 2011 found that only a small percentage of pregnant women received skilled laborers' antenatal care (ANC) (34%), institutional delivery (10%), and postnatal care (PNC) (6%) services, which led to high rates of maternal and neonatal morbidity and mortality [16]. The adoption level of mHealth in resource-limited settings was low [1, 16]. The situation, the innovation, the business model, the technology adoption system, the institutional infrastructure, the background, and the communication between each of these disciplines influence the successful introduction of new technological innovations [17]. Evidence also suggests that the success of a mHealth initiative is dependent on its accessibility, acceptance, effective adaptation to local contexts, and strong stakeholder collaboration [18].

To launch the latest initiatives, it is also important to take into account the multicultural environment, which contains societal and cultural disparities [19]. Among the numerous factors that help ensure the successful implementation of mHealth interventions, the end-user perspective and the value of the new system are essential to take into account before actual implementation [14, 20]. The nation has recently paid close attention to mobile health services due to unresolved issues in the healthcare system. Additionally, one of Ethiopia's four transformation agendas is the health information revolution, which primarily focuses on delivering excellent health services through evidence, access to health services, and equity. As a result, mHealth acceptance, use, and overcoming barriers to use are becoming increasingly crucial. Moreover, as per our knowledge, a previous study on attitudes toward mHealth was conducted among patients, so no study has been conducted on healthcare professional's attitudes to using mHealth in southwest Ethiopia. To fill the gap, this study is aimed at assessing healthcare professionals' attitudes and associated factors toward mHealth in resource-limited settings.

## 2. Methods and Materials

### 2.1. Study Design, Period, and Area

An institution-based cross-sectional study was carried out among healthcare professionals in southwest public hospitals. The study was carried out in public facilities in Ilu Abba Bor and Buno Bedelle zones, Oromia regional state, southwest Ethiopia. Illu Abba Bor and Buno Bedelle Zones are the Oromia regional state situated southwest of the region and located at a distance of about 600 km and 483 km from the center of the region, respectively. In the two zones, there are five public hospitals, namely, Bedele, Darimu, Dembi, Metu Karl, and Chora Hospital. The study was conducted from January 08 to February 10, 2023.

### 2.2. Study Population

All healthcare professionals who worked in Oromia regional state hospitals were the source of the population. Healthcare professionals including general practitioners, residents, specialists, and subspecialists who were working in the Oromia regional state of public hospitals during the study period were the study population. All healthcare professionals who had served at least 6 months before the study were included in the study. All healthcare professionals who were on annual leave, sick leave, and who left for a long-time education were excluded from the study because those participants might have difficulty responding to organizational-related questions, which might influence their attitude toward using mobile health technology.

### 2.3. Study Variables and Measurements

The outcome variable was the attitude to using mobile health technologies, sociodemographic characteristics, and organizational factors, which were assessed using six yes and no questions [21], and access technology and mobile device utilization patterns assessed using four-item questions [21, 22] were considered predictor variables for the outcome of the variables in this study. The attitude of the respondents was assessed by using an item rated on eight questions of a five-point Likert scale that ranged from “1 = *strongly disagree*” to “5 = *strongly agree*.” The scores of the Likert scale statement were dichotomized into two. In this study, a score of an individual's response less than ≤ 3 was labeled as “unfavorable or negative” and greater than 3.0 was labeled as a “favorable or positive” attitude [23].

### 2.4. Sample Size Determination and Sampling Procedure

The sample size was determined using the single population proportion formula by the following assumptions:

 $$\begin{array}{c} n=\frac{{\left(Z\;\alpha /2\right)}^{2}\times p\left(1-p\right)}{d^{2}}=\frac{{\left(1.96\right)}^{2}\times 0.5\left(0.5\right)}{{\left(0.05\right)}^{2}}=384 \end{array}$$

After we consider the nonresponse rate of 10%, finally, 384 + 384(0.1) = 422, where *n* is the estimated sample size, *p* is the single population proportion (50%) because the attitude toward mobile health technology in Ethiopia was not investigated, *Z*/2 is the 95% level of confidence interval, and *d*^2^ is the 5% margin of error.

To select the study participants, first, we received the sample unit from the hospital management, and the entire sample size was proportionally allocated according to the number of healthcare professionals in each hospital. Finally, a systematic random sampling technique was used to recruit the participants in the specified hospitals.

### 2.5. Data Collection Tool, Data Quality Control, and Procedure

Data were gathered using standardized, pretested, interviewer-administered questionnaires that were adapted from available research [21, 23–25]. To evaluate the validity and reliability of the data collection instrument before the actual data collection, a pretest was conducted outside of the study setting, which was in Jimma Hospital, with 10% of the total sample size, and necessary modifications were made accordingly. Cronbach's alpha was used to evaluate the internal consistency for each aspect of the data collection instrument. Finally, for the actual data collection, 2 days of training was provided for three nursing professionals, three health informatics professionals who were data collectors, and three supervisors.

### 2.6. Data Processing and Analysis

Data entry was done using Epi Data Version 4.6, and analysis was done using STATA Version 14. For descriptive statistics, frequencies and percentages were determined and presented using graphs and tables. A binary logistic regression model was used to identify variables that are significantly associated with the outcome predictor. To control the possible effects of confounders, variables with a *p* value of less than 0.2 in the bivariable logistic regression analysis were entered into the multivariable logistic regression analysis. Both crude odds ratio (COR) and adjusted odds ratio (AOR) with 95% confidence intervals were computed to show the strengths of associations [14]. Finally, a *p* value of less than 0.05 at the multivariable logistic regression analysis was used to identify variables significantly associated with the attitude of health professionals to using mobile health technology.

The Cronbach alpha measured the reliability/consistency of the data, and the value was above 0.84. The variance inflation factor (predictor VIF < 0.35) was used with a cut-off point of 10 to determine whether multicollinearity existed among independent variables, and there was no evidence of it. Finally, the model fit was examined using the Hosmer and Lemeshow goodness-of-fit test (*p* value = 0.27).

## 3. Result

### 3.1. Sociodemographic Characteristics of Healthcare Professionals

A total of 415 health professionals were enrolled in this study, with a response rate of 98.3%. The majority of the participants (257 [61.9%]) were males. The mean age of the respondent was 29.6 ± 5.3 SD years. Regarding the specialty, 162 (39%) of the study participants were nurse professionals. In terms of educational status, almost above half (237 [57.1%]) of the study participants had bachelor's degrees. One hundred ninety-four (46.7%) of the respondents had 3 and below years of work experience. Moreover, the majority of the respondents (267 [64.3%]) had between 5000 and 10,000 monthly income (Table 1).

### 3.2. Access to Basic Technologies and Patterns of Usage Among Healthcare Professionals

Of the study participants, almost three-fourths (311 [74.7%]) of the respondents had mobile devices. Moreover, the majority of 391 (94.3%) of the respondents used social media; however, 386 (93%) of the respondents did not interact with patients through social media (Table 2).

### 3.3. Organizational-Related Variable on Attitudes Toward mHealth Technologies Among Healthcare Professionals

The finding revealed that more than three-fifths of 270 (65.1%) of the study participant had access to the internet. Two hundred fifty-one (60.5%) of the respondents had taken training about mobile health technology. Concerning ICT infrastructure, almost half (201 [48.4%]) of the respondents showed good ICT infrastructure in the organizations. Two hundred ninety-nine (72.4%) of the study participants could be obtained from ICT technical support. In addition, 255 (61.4%) of respondents took computer-related training (Table 3).

### 3.4. Attitude Toward Mobile Health Technology Among Healthcare Professionals

**Figure 1:**
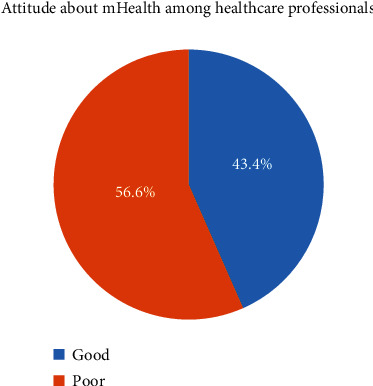
Attitude toward mHealth technology in southwest public hospitals, Ethiopia, 2023 (*n* = 415).

Among the total of 415 health professionals, 180 (43.4%) respondents had a favorable attitude toward mHealth technology in southwest public hospitals (Figure 1).

### 3.5. Factors Associated With the Attitude of Healthcare Professionals Toward mHealth Technologies in Southwest Public Hospitals

The bivariate analysis findings revealed that gender, educational level, knowledge about mHealth, work experience, ICT infrastructure, own mobile phone, computer training, and internet access in the organization were associated with attitude to using mHealth technologies at a *p* value of < 0.2. All of these associated factors were entered into the multivariable logistic regression analysis model to control for the effect of confounders. Finally, the multivariable logistic regression model identified that educational level (master and above), good knowledge, work experience (≥ 5 years), ICT infrastructure, had own smart mobile phone, and computer training were associated with attitude to using mHealth technologies at a *p* value of < 0.05 (Table 4).

As the result summarized in Table 4 shows, healthcare professionals with master's degrees and above were 3.67 times more likely to be willing to use mobile health technologies (AOR: 3.67; 95% CI: 1.22, 4.10) than those who had a bachelor's degree and below educational level after controlling other variables. Healthcare professionals who had good knowledge of mobile health technology were 4.08 times more likely to be willing to use mobile health technologies (AOR: 4.08; 95% CI: 1.35, 5.31) than their counterparts, keeping other variables constant. Healthcare professionals who had more than 5 years of work experience were 3.09 times more likely to be willing to use mobile health technologies (AOR: 3.09; 95% CI: 1.76, 5.60) as compared with their counterparts. Similarly, healthcare organizations that had ICT infrastructure were 2.70 times more likely to be willing to use mobile health technologies by healthcare professionals (AOR: 2.70; 95% CI: 1.38, 5.31).

Additionally, healthcare professionals who had their smart mobile phones were 3.67 times more likely to be willing to use mobile health applications (AOR: 3.67; 95% CI: 3.20, 4.31) as compared with healthcare professionals who did not have their smart mobile phone. Similarly, healthcare professionals who had taken computer-related training were 1.96 times more likely to be willing to use mobile health technologies (AOR: 1.96; 95% CI: 1.03, 3.73) as compared with healthcare professionals who did not take computer training (Table 4).

## 4. Discussion

### 4.1. Principal Findings

The findings of this study revealed that 43.4% (95% CI: 39.6–48.3) of the study participants had a favorable attitude to using mHealth technology at public hospitals in southwest Ethiopia. This finding was lower than the study conducted in Ethiopia [22], and 64% of respondents had a good attitude toward telemedicine. This discrepancy might be due to the variation between study participants on sample size, study area, and the intent of researchers toward the scope of their study which is they are going to give more emphasis, and there is a continuous computer and other related training gap among the study participants across the study area.

And also, the finding is lower than the studies conducted in Nigeria [15]. A total of 97.6% of study participants had a positive attitude to the use of mHealth in health practice in Kuwait [26], 69.4% of the study participants had a positive attitude toward eHealth in Libya [27], 82.6% of healthcare workers reported a high attitude toward telemedicine, and only 30% of the study participants had a low attitude toward telemedicine in India [28]. This difference might be due to differences in countries' ICT infrastructure, internet access, and technology use, as well as variation between study participants. As a result, the finding showed that there is still more to be done in terms of mobile health technology education for healthcare providers in order to set the stage for a successful and long-lasting uptake of the technology in the nation.

According to our findings of job experience of the study, participants were positively associated with their attitude toward mHealth technology. This finding goes with the study conducted in Nigeria [15] and Ethiopia [23] about attitudes to the use of information communication technology. The possible reason for this fact is that those who stay on their job for a long time initiate them to improve their professional skills with the help of new technology; so, this might increase their attitude toward using new emerging technologies. However, a study done in Kenya showed that the physicians' prior experience with the previously deployed EMR system and their attitude about the system were shown to be inversely related [14]. The prior EMR system may have been the reason because it was poorly designed and used nonstandard medical terminology. Educational status was positively associated with study participants' attitudes toward mHealth. This finding was consistent with studies conducted in Ghana [29]. This may be explained by the fact that less-educated individuals have lower levels of attitude about new technology like mobile-based and telehealth-related technology as compared to those with the highest educational level. Also, according to our study findings, study participants who had master's degrees and above educational level were more likely to have good attitudes than their counterparts. The possible reason was that as individuals learn at a higher educational level, they might have a chance to access different websites, journal articles, and different technologies and participate in different online meetings, e-learning, etc. So, through this process, they may develop a positive attitude toward new emerging technologies in the area of the health profession.

The knowledge of healthcare professionals toward mobile health technology was significantly associated with the attitude toward using the technology. This finding was consistent with previous studies on health providers' attitudes to the use of telerehabilitation in Ethiopia [21], Saudi Arabia [30], and Tehran [31]. Another study conducted in Pakistan showed that healthcare professionals' knowledge influences their attitude to using information technology [32]. The possible reason could be that knowledge of health technology improved their attitude to using digital technologies in healthcare.

In this study, being an owner of their smartphone was positively associated with study participants' attitudes toward mHealth care. This finding is in line with a study conducted in Saudi Arabia [33] and Bangladesh [34]. This fact might be due to those individuals who had a computer like a smartphone and other related technology being more likely to have a positive attitude toward using new advanced technology since they have a chance to visit different websites and social media by using their smartphone [33].

Additionally, the usage of smart phones can enhance patient-provider communication, which could enhance patient management procedures as a whole. As a result, health providers may develop a good attitude toward using new advanced technology in their profession.

ICT infrastructure in an organization was significantly correlated with participants' attitudes toward mHealth technology. Participants who reported that their organization had a good ICT infrastructure outperformed those who did not by a factor of 2.7 (AOR = 2.70; 95% CI: 1.38–5.31) and were more likely to have a positive attitude toward mHealth. This finding was consistent with research done in Ethiopia [23] about attitudes to the use of information communication technology. A possible explanation might be that the availability of ICT infrastructure emphasizes the attitude to using mobile health technology. However, it was inconsistent that the study was done in Saudi Arabia [35]. In this study, the ICT infrastructure of the organization was not a matter for the users. This means that, in Saudi Arabia, there is better ICT infrastructure, which is equally distributed among study participants as compared to our country. In that way, ICT infrastructure did not affect their attitudes.

In our study, computer training was positively associated with study participants' attitudes toward mHealth care technology. Among the study participants, those who had computer training were 1.96 times (AOR = 1.96; 95% CI: 1.03–3.73) more likely to have a good attitude about mHealth care technology than those individuals who had no computer training. This finding was consistent with a study conducted in Saudi Arabia [35], India [36], and Ethiopia [37]. This consistency might be because continuing and regular technology-related training increases individuals' attitudes toward new technology use. Also, evidence shows that continuous training is necessary for the use of new emerging technology [35]. Moreover, another possibility is that computer training was more likely to increase respondents' familiarity in using technologies, and the best way to teach healthcare professionals how to effectively address the opportunities and problems presented by mobile health technologies is through well-organized training.

### 4.2. Limitations of the Study

This study has a few limitations. The study was conducted using only a quantitative approach. In addition, the study was conducted only in public hospitals, which may affect the generalizability of the findings to other settings. Due to the nature of the study, which used a self-administered questionnaire, participants might have been exposed to social desirability or response bias. Moreover, the associations between the variables were measured using a cross-sectional design, which may not provide well-established casualties. Future works would be better to incorporate a qualitative approach and extend the study settings to have more strength in the findings.

## 5. Conclusion

Overall, healthcare professionals' attitude to using mobile health technologies in southwest Ethiopia was relatively low. The highest educational level, good knowledge, more than 5 years of work experience, ICT infrastructure, having a smart mobile, and having taken computer-related training were significant factors associated with healthcare professionals' attitude to using mobile health technologies. Considering these factors could provide insight into developing and adopting mobile health technologies in resource-limited settings.

## Figures and Tables

**Table 1:** Sociodemographic characteristics of healthcare professionals in southwest public hospitals, Ethiopia, 2023 (*n* = 415).

**Variables**	**Category**	**Frequency**	**Percentage (%)**
Gender	Male	257	61.9
	Female	158	38.1

Age (mean ± SD)		29.6 ± 5.3	

Profession	Nurse	162	39.0
	Laboratory	39	9.4
	Midwifery	50	12.0
	Doctor	121	29.2
	Other health professionals	43	10.4

Educational status	Bachelor and below	237	57.1
	Master and above	178	42.9

Work experience	≤ 3	194	46.7
	3–5	65	15.7
	≥ 5	156	37.6

Monthly income	≤ 5000	107	25.8
	5000–10,000	267	64.3
	≥ 10,000	41	9.9

*Note:* Others: physiotherapist, health officer, psychiatrist, pharmacist, optometrist, and anesthesia.

**Table 2:** Access to basic technologies and pattern of usage among healthcare professionals in southwest public hospitals, Ethiopia, 2023 (*n* = 415).

**Variables**	**Category**	**Frequency**	**Percentage (%)**
Do you have a mobile device?	Yes No	402 13	96.8 3.2
Do you have used social media?	Yes No	391 24	94.3 5.7
Which social media is usually used?			
Facebook	Yes No	310 81	79.3 20.7
Telegram	Yes No	202 189	51.7 49.3
Twitter	Yes No	139 252	35.6 64.4
Instagram	Yes No	141 250	36.1 63.9
Other	Yes No	169 222	43.2 56.8
In your role, do you interact with patients through social media on your mobile devices?	Yes No	29 386	7.0 93.0

**Table 3:** Organizational-related variable on attitudes toward mHealth technologies among healthcare professionals in southwest public hospitals, Ethiopia, 2023 (*n* = 415).

**Variables**	**Category**	**Frequency**	**Percentage (%)**
Good internet access?	Yes	270	65.1
	No	145	34.9
Training for mHealth	Yes	251	60.5
	No	164	39.5
ICT infrastructure in your organization	Yes	201	48.4
	No	214	51.6
ICT technical support	Yes	299	72.4
	No	116	27.6
Computer-related training	Yes	255	61.4
	No	160	38.6

**Table 4:** Bivariate and multivariate logistic regression factors associated with the attitude of healthcare professionals toward mHealth technologies in southwest public hospitals, Ethiopia, 2023 (*n* = 415).

**Characteristics**	**Favorable attitude,** **n** **(%)**	**Unfavorable attitude,** **n** **(%)**	**COR (95% CI)**	**AOR (95% CI)**
*Gender*				
Female	98 (62.1)	60 (37.9)	1	1
Male	150 (58.4)	107 (41.6)	1.17 (1.09, 1.81)	1.13 (0.66, 2.43)
*Educational level*				
Bachelor and below	145 (61.2)	92 (38.8)	1	1
Master and above	99 (55.6)	79 (44.4)	1.26 (1.21, 2.99)	3.67 (2.06, 6.55)^∗∗^
*Knowledge*				
Good	123 (49.6)	125 (50.4)	1.02 (1.24, 2.33)	4.08 (1.35, 5.31)^∗^
Poor	82 (49.1)	85 (50.9)		1
*Work experience*				
≤ 3	100 (51.5)	94 (48.5)	1	1
3–5	36 (55.4)	29 (44.6)	0.86 (0.65, 0.99)	1..44 (1.77, 2.70)
≥ 5	76 (48.7)	80 (51.3)	1.12 (1.04, 3.12)	3.09 (1.76, 5.60)^∗^
*ICT infrastructure*				
Yes	103 (51.2)	98 (48.8)	1.15 (1.01, 2.97)	2.70 (1.38, 5.31)^∗∗^
No	102 (47.7)	112 (52.3)	1	1
*Own smartphone*				
Yes	188 (49.6)	191 (50.4)	0.91 (0.23, 0.96)	3.67 (3.20, 4.31)^∗^
No	17 (47.2)	19 (52.8)	1	1
*Computer training*				
Yes	123 (48.2)	132 (51.8)	1.13 (1.14, 3.87)	1.96 (1.03, 3.73)^∗∗^
No	82 (51.2)	78 (48.8)	1	1
*Internet access*				
Yes	130 (48.1)	140 (51.9)	1.15 (1.02, 2.98)	0.71 (0.31, 1.64)
No	75 (51.7)	70 (48.3)	1	1

^∗∗^ *p* value < 0.01.

^∗^ *p* value < 0.05.

## Data Availability

Data will be available upon reasonable request from the corresponding author.

## Nomenclature

AOR: adjusted odds ratio
CI: confidence intervals
COR: crude odds ratio
ICT: information communication technology
STATA: Statistical software
WHO: World Health Organization
